# Evaluating medication discrepancies and harm: a matched cohort study of collaborative pharmacist prescribing in a statewide healthcare system using electronic prescribing

**DOI:** 10.1007/s11096-025-01996-y

**Published:** 2025-08-28

**Authors:** Hana Amer, Sally Marotti, Joshua M. Inglis, Imaina Widagdo, Sharon Goldsworthy, Jacinta Johnson, Lisa Kalisch Ellett

**Affiliations:** 1 Quality Use of Medicines and Pharmacy Research Centre, UniSA Clinical and Health Sciences, University of South Australia, GPO Box 2471, Adelaide, SA 5001 Australia; 2 SA Pharmacy, Adelaide, SA Australia; 3 Department of Clinical Pharmacology, Flinders Medical Centre, Flinders University, Bedford Park, SA Australia; 4 School of Biomedicine, Faculty of Health Sciences, University of Adelaide, Adelaide, SA Australia

**Keywords:** Electronic prescribing systems, Interprofessional collaboration, Non-medical prescribing, Pharmacist prescribers, Prescription errors

## Abstract

**Introduction:**

Collaborative pharmacist prescribing models involve pharmacists working with doctors and patients to develop medication plans and prescribe medications. Limited evidence exists on the impact of these models on medication discrepancies in hospitals using electronic prescribing systems (EPS).

**Aim:**

This study aimed to evaluate the impact of collaborative pharmacist prescribing on medication discrepancies and potential patient harm within a statewide healthcare system using EPS.

**Method:**

A multi-site matched cohort study involving 240 patients was conducted. EPS data for 120 patients aged ≥ 18 years who received collaborative pharmacist prescribing was matched 1:1 with 120 patients who received usual care of independent medical prescribing. Matching variables were hospital, clinical unit, sex, age, admission date, triage category and pre-admission medication count. The electronic medical record was reviewed to identify undocumented medication discrepancies, which were defined as any unexplained difference between the pharmacist-led medication history and medications prescribed on admission. The frequency of undocumented discrepancies was calculated. An independent multi-disciplinary clinician panel determined potential harm, using the Harm Associated with Medication Error Classification (HAMEC) tool.

**Results:**

There were fewer undocumented discrepancies per medication prescribed in the collaborative pharmacist prescribing group compared to usual care (RR 0.04, 95% CI 0.03–0.06) and the relative risk of undocumented discrepancies per patient was lower (RR 0.23, 95% CI 0.13–0.39). The expert clinician panel found that undocumented discrepancies rarely posed serious or severe harm in either group (0 undocumented discrepancies with potential to cause serious or severe harm in the collaborative pharmacist prescribing group compared to 8 in the usual care group).

**Conclusion:**

The implementation of collaborative pharmacist prescribing within a statewide EPS significantly reduced undocumented discrepancies and lowered the potential for patient harm. As healthcare systems globally shift towards electronic prescribing, this study provides timely and actionable evidence to inform policy and support the adoption of collaborative prescribing models in hospitals using EPS. Such models offer a practical strategy to improve medication safety, reduce patient harm and strengthen interprofessional collaboration at the point of prescribing.

**Supplementary Information:**

The online version contains supplementary material available at 10.1007/s11096-025-01996-y.

## Impact statements

- Collaborative pharmacist prescribing in hospitals utilising electronic prescribing systems significantly reduces medication discrepancies and potential patient harm compared to usual care
- These findings support the integration of collaborative pharmacist prescribing in electronic prescribing environments to improve medication safety, patient harm, and to strengthen interprofessional collaboration at the point of prescribing
- Given the global shift towards electronic prescribing, this evidence is timely and relevant for guiding healthcare organisations and policymakers seeking to enhance quality of care
- These findings support broader international efforts to expand pharmacist scope of practice through collaborative prescribing to ease workforce pressures without comprising on quality and safety

## Introduction

Pharmacist prescribing has been embedded within community and hospital settings in many countries internationally, with three broad models commonly used: independent, dependent and collaborative (or supplementary) pharmacist prescribing [1]. Independent prescribing has been implemented in the UK since 2006 [2], and Alberta (Canada) since 2007 [3], with pharmacists leading the patient assessment, diagnosis and prescribing [1]. Dependent prescribing models have been implemented in some states in the US [4], and in Australian community pharmacies [5], where pharmacists prescribe medications from a restricted list for specific indications according to a protocol, guideline, or formulary [1]. Collaborative prescribing models have been adopted in other jurisdictions in the US [6] and Australia in hospital [7–15] and general practice [16, 17] settings. In the UK, whilst independent prescribing has become the predominant model, collaborative models are still being used in multi-disciplinary settings due to the team-based nature of care delivery [18–21]. Collaborative prescribing models typically require the initial patient assessment and diagnosis to be made by a doctor, with the doctor and pharmacist working together to develop an agreed medication plan, and the pharmacist prescribing the medications accordingly [1, 22]. The medication plan is developed collaboratively using the pharmacist-led medication history [23], working diagnosis and relevant laboratory and diagnostic test results [22]. Some collaborative models require that medications prescribed by a pharmacist are reviewed and countersigned by a treating doctor before the medications can be administered to the patient [22]. In other collaborative models, pharmacists can prescribe medications for administration, without a countersignature [22].Collaborative pharmacist prescribing models implemented in paper-based prescribing systems have been shown to significantly reduce prescribing error rates from 66% of patients having one or more error with independent medical prescribing to 3.6% with collaborative pharmacist prescribing [24]. Collaborative pharmacist prescribing has also been shown to reduce median length of stay by 10% (from 6.5 to 5.8 days) and the average cost of admissions by $726 (from $9,803 to $8,802) [25].

The majority of evidence relating to the benefits of collaborative pharmacist prescribing comes from settings where paper-based prescribing systems are used [7–10, 15]. Broader research on electronic prescribing systems (EPS) indicates that EPS can reduce procedural errors such as unclear or incomplete orders but have less impact on clinical errors such as medication omissions [26]. A systematic review conducted in 2017 [1] identified seven papers on collaborative pharmacist prescribing models in the hospital setting: five studies from Australia and two from the US were included. Most of these collaborative models were implemented in outpatient or ambulatory care settings, with only two implemented in the inpatient hospital setting [1]. No studies were reported to be implemented in electronic settings [1]. Five out of seven models required a medical countersignature for pharmacist prescriptions [1].

Emerging research suggests potential benefits of collaborative pharmacist prescribing in hospitals using EPS. For example, a pre-and-post study of orthopaedic patients found that the median number of medication errors on admission declined from six to one per patient after collaborative pharmacist prescribing was implemented (*p* < 0.01) [14]. Another study in an acute medical unit using EPS, reported a reduction in average admission errors from 4.41 to 0.52 errors per patient (*p* < 0.0001) and 0.43 to 0.05 errors per medication prescribed (*p* < 0.005) with collaborative pharmacist prescribing [12]. A randomised trial conducted in a hospital geriatric unit compared collaborative pharmacist prescribing on discharge to usual care practice of independent medical prescribing for both handwritten prescriptions and digital prescribing [11]. The study demonstrated a reduction in prescribing errors with collaborative prescribing compared to usual care (RR 0.31, 95% CI 0.16–0.58, *p* < 0.0002) [11]. There was no significant change in the error rate between handwritten or digital prescriptions [11]. In these studies, pharmacists prescribed medications ‘on hold’ pending electronic countersignature and release by a treating doctor. These studies were all conducted within single hospitals, with two of the studies [11, 14] involving only a single prescribing pharmacist. The generalisability of findings across models with different countersignature requirements, prescribers, patient cohorts and hospitals remains unclear. With the growing use of electronic medical records, and prescribing models moving away from requiring medical countersignatures, it is increasingly important to understand the impact of collaborative pharmacist prescribing models in these settings.

### Aim

This study aimed to evaluate the impact of collaborative pharmacist prescribing on medication discrepancies and potential patient harm within a statewide healthcare system using electronic prescribing. It also examined whether the accuracy of pharmacist prescribing changed when medical countersignature requirements were removed.

## Method

### Study setting and collaborative pharmacist prescribing models of care

Collaborative pharmacist prescribing was implemented in acute and general medical units at three South Australian public hospitals in a staggered rollout between March and October 2022. Pharmacists obtained and documented a medication history on admission (pharmacist-led medication history). Pharmacists then worked with doctors and patients to develop an individualised medication plan, documented in the electronic medical record (EMR) and signed by both the pharmacist and doctor. The pharmacist could prescribe medications ‘on hold’ in the EPS according to the plan with a doctor required to electronically countersign and ‘release’ medications for administration. In late 2023, a revised pathway allowed pharmacists to prescribe medications listed on the plan, without requiring medical countersignature. The models were otherwise identical, differing only in the requirement for medication release. In this study the term ‘collaborative pharmacist prescribing’ is used to describe both models, regardless of whether a medical countersignature was required. Pharmacist prescribers across all sites had a minimum of 2-years hospital clinical pharmacy experience and successfully completed the same collaborative prescribing training program. All three sites used the same EMR which incorporated electronic prescribing, (Sunrise™), allowing for consistent evaluation of the model across hospitals.

### Study design and participants

A multi-site matched cohort study was conducted. At each of the three participating hospitals patients aged ≥ 18 years, admitted to an acute or general medical unit with a pharmacist prescriber between 1st February 2023 and 5th March 2024, and had a pharmacist-led medication history within 48 h of the admission, were eligible to receive collaborative pharmacist prescribing. Due to staffing constraints, not all patients in acute or general medical units with a pharmacist prescriber received the collaborative prescribing service. Pharmacists prioritised high-risk patients to receive the service, like they do for standard pharmacy services, including patients with a formal medical team referral or those at high-risk of medication-related problems, suspected medication-related admissions, age ≥ 65 years, polypharmacy (≥ 5 medications), or use of a high-risk medication [23]. Participants in the usual care group were eligible for inclusion if they were admitted to a clinical unit where collaborative prescribing was implemented, had a pharmacist-led medication history within the first 48 h of their admission, and had their medications prescribed by a doctor as per usual practice. To mitigate potential bias due to non-random service allocation, we case-matched patients in the usual care group to those who received collaborative prescribing.

Pharmacist-led medication histories have been shown in prior research to provide a ‘gold standard’ [27–29] for medications taken pre-admission. In the usual care group, doctors prescribed medications independently, and in many cases, did so before the pharmacist-led history had been taken. This is standard care at the hospitals in our study.

Matching was exact for hospital site and unit, with closest matches for sex, age at admission, date of admission, triage category and pre-admission medication count. Randomisation and matching were performed using the RStudio MatchIt package (version 1.4.543). Patients who received collaborative pharmacist prescribing were identified in the EMR by the patient medication plan which was unique to the collaborative prescribing model. Furthermore, medication orders listed the prescriber type, enabling orders prescribed by a pharmacist to be distinguished from those prescribed by doctors. One hundred and twenty patients who received collaborative pharmacist prescribing were randomly selected and matched 1:1 to 120 patients who received independent medical prescribing (usual care).

### Data collection

An independent pharmacist researcher extracted patient information from the EMR. Pre-admission medications were identified from the pharmacist-led medication history, including both regular and ‘as required’ (prn) medications. Medications prescribed within 24 h of admission were also extracted from the EMR, using EPS prescribing timestamps to identify medications prescribed in this timeframe. The independent pharmacist researcher identified discrepancies between the pharmacist-led medication history and medications prescribed within 24 h of the admission. This method has been used in previous studies which have compared pharmacist and medical prescribing [7, 12, 24].

Discrepancies were classified using an adapted MedTax taxonomy [30]: omitted medication, incorrect dose, incorrect frequency, incorrect medication, unnecessary medication, incorrect administration route, incorrect administration time, incorrect formulation, or other discrepancy. All discrepancies were independently reviewed by two additional pharmacist researchers blinded to study group, with disagreements resolved through consensus. Recorded reasons for discrepancies were retrieved from the EMR; those without a documented reason were classified as ‘undocumented.’

An independent expert panel of two senior pharmacists and two senior physicians assessed all undocumented discrepancies for potential harm, using the *Harm Associated with Medication Errors Classification (HAMEC) tool* [31]. *HAMEC* assumes the error reached the patient undetected, with potential harm rated from level 0 (no harm) to level 4 (severe harm) [31] (see Supplementary Table 2 for HAMEC harm definitions). Panel members were blinded to patient group and site. Following HAMEC guidelines [31], panel members received the medication name, dose, route, frequency (including whether regular or prn), and type of MedTax discrepancy [30]. Panel members were provided with an information sheet detailing the steps of performing the harm assessment using the HAMEC tool [31], including example cases, and were trained on how to use the tool to improve inter-rater reliability. Using a modified Delphi method [32], panel members independently rated potential harm. Discrepancies with < 75% agreement or a > 1-point difference in ratings (n = 230 of 449 total discrepancies) were discussed in a meeting with all panel members to reach full consensus.

For patients in the collaborative pharmacist prescribing group, an additional analysis was conducted to identify discrepancies between new medications listed in the medication plan and medications prescribed within the first 24 h of admission. Participants in the usual care group were not included in this, as they did not receive a medication plan. These additional discrepancies were classified using the same method described previously.

### Outcome measures

The primary outcome was the frequency of undocumented medication discrepancies in each group, expressed as the proportion of patients with one or more undocumented medication discrepancies and the number of undocumented medication discrepancies as a proportion of all medications prescribed.

Secondary outcomes included the frequency of each type of undocumented medication discrepancy and the potential for patient harm associated. A sensitivity analysis was conducted to determine if there was a difference in the frequency of undocumented discrepancies in the collaborative pharmacist prescribing group in the period when medical officer countersignature was required, compared to the period when the requirement for medical officer countersignature prior to administration was removed. The staggered rollout of models across the different hospital sites determined the exact timeframes of when the countersignature was and wasn’t required, as shown in Supplementary Fig. 1.

### Data analysis

Continuous skewed data were reported as medians (interquartile range, IQR) and categorical data as counts and percentages. Group comparisons used the Mann–Whitney U test for medians and Fisher’s exact test for cell counts less than five. Relative risk (RR) and relative risk reduction (RRR) with 95% confidence intervals (CIs) were calculated. Statistical significance was defined as *p* < *0.05*. Analyses were performed using SPSS (IBM Corporation, Armonk, NY, USA).

### Sample size

A minimum of 18 patients per group per site (total 108) was required, based on 95% confidence, 80% power, and expected discrepancy rates of 50% with usual care [10] versus 9% with collaborative pharmacist prescribing [33]. A larger sample (240 patients) was used, as prior estimates were based on paper-based systems and there was an expectation that the frequency of discrepancies may differ with electronic prescribing [14]. A post-hoc power calculation confirmed 100% power to detect a statistically significant difference for the primary outcome.

### Ethics approval

Approval to conduct the study was obtained from the Central Adelaide Local Health Network Human Research Ethics Committee (approval number: 16945, approved 08/11/2022) and the University of South Australia Human Research Ethics Committee (approval number: 205819, approved: 07/09/2023).

## Results

During the study period, 59,140 patients were admitted to acute or general medicals unit across the three hospitals and met inclusion criteria. Of these, 2,146 received collaborative pharmacist prescribing and 56,994 received usual care. From this population, 240 patients were randomly selected for inclusion in the study, 120 in the collaborative pharmacist prescribing group and 120 in the usual care group. No outcome data were missing, and all 240 patients were included in the final analysis. Half of the collaborative pharmacist prescribing patients (n = 60) were from the period requiring medical countersignature, and half from the period when it was no longer required (Supplementary Fig. 1). Groups were successfully matched, with no statistically significant differences between groups for matching variables (Supplementary Table 1).

Participants had a median age > 75 years, and over half were female (Table 1). All patients had a pharmacist-led medication history within 48 h of admission. Fourteen (12%) in the usual care group had this before medications were prescribed, compared to 100% in the collaborative pharmacist prescribing group (*p* < 0.001). Patients in the collaborative pharmacist prescribing group had a higher median number of medications prescribed after admission compared to the usual care group (15 vs 11, *p* < 0.0001) (Table 1).

**Table 1:** Patient demographics

	Collaborative pharmacist prescribing group (n = 120 patients)	Usual care group (n = 120 patients)	*p*
Age (y) Median (IQR)	75 (65–85)	78 (64–85)	0.634
Female sex, n (%)	67 (56%)	66 (55%)	0.897
Previous residence, n (%)			
Private residence	100 (83%)	95 (80%)	0.447
Supported accommodation (low- and high-level residential aged care facilities, domestic supported living facilities and independent units as part of a retirement village), n(%)	20 (17%)	25 (20%)	
Pharmacist-led medication history completed before prescribing, n (%)	120 (100%)	14 (12%)	< 0.001
Medications prescribed within 24 h of admission, median (IQR)^a^	15 (11–19)	11 (6–16)	< 0.001

^a^Patients were matched based on the number of medications prescribed prior to admission; however the number of medications prescribed on admission was not a matching variable

Pharmacists prescribed 1900 medications in the collaborative pharmacist prescribing group, with 25 undocumented discrepancies (1% of medications prescribed). In the usual care group, 1339 medications were prescribed, with 424 discrepancies (32% of medications prescribed) (*p* < 0.001). The relative risk of an undocumented discrepancy per medications prescribed was reduced by 96% in the collaborative pharmacist prescribing group compared to usual care (Table 2). Thirteen of the 120 patients (21%) in the collaborative pharmacist prescribing group had at least one undocumented discrepancy, compared to 108 patients (90%) in the usual care group. The relative risk of a patient having at least one undocumented discrepancy was reduced by 77% in the collaborative prescribing group compared to usual care (Table 2).

**Table 2:** Undocumented medication discrepancies each group

	Collaborative pharmacist prescribing group	Usual care group	Relative risk (95% confidence interval)
Total medications prescribed	1900	1339	0.04 (0.03–0.06)
Undocumented discrepancies (% of medications prescribed)	25 (1%)	424 (32%)	
Total patients	120	120	0.23 (0.13–0.39)
Patients with one or more undocumented discrepancy (% of patients)	13 (21%)	108 (90%)	
Number of discrepancies (% of patients)			N/A
0	95 (80%)	12 (10%)	
1	23 (19%)	12 (10%)	
2	0 (0%)	26 (22%)	
≥ 3	2 (2%)	70 (58%)	

Additional analysis in the collaborative pharmacist prescribing group showed that there were no undocumented discrepancies between the medication plan and medications prescribed within 24 h of admission.

Medication omissions were the most common undocumented discrepancies in both groups (84% in the collaborative pharmacist prescribing group and 73% in usual care) (Table 3).

**Table 3:** Types of undocumented medication discrepancies

Type of undocumented medication discrepancy	Collaborative pharmacist prescribing group (n = 25 undocumented discrepancies)	Usual care group (n = 424 undocumented discrepancies)
Omitted medication	21 (84%)	309 (73%)
Incorrect dose	2 (8%)	60 (14%)
Incorrect time of administration	1 (4%)	26 (6%)
Unnecessary medication	0	13 (3%)
Incorrect frequency	0	8 (2%)
Incorrect medication	1 (4%)	3 (0.7%)
Incorrect formulation	0	4 (1%)
Other	0	1 (0.2%)

### Potential harm associated with undocumented discrepancies

The clinical panel determined that over 80% of discrepancies in both groups had the potential to cause no or minor harm. Eight discrepancies (2%) in the usual care group and no discrepancies in the collaborative pharmacist prescribing group were assessed as having the potential for severe or serious harm (Table 4). Examples of undocumented discrepancies and their potential for patient harm as assessed by the expert panel are shown in Supplementary Table 3.

**Table 4:** Potential for patient harm associated with undocumented medication discrepancies

HAMEC score	Collaborative pharmacist prescribing group (n = 25 undocumented discrepancies)	Usual care group (n = 424 undocumented discrepancies)
Level 0: No harm	9 (36%)	175 (41%)
Level 1: Minor harm	13 (52%)	181 (43%)
Level 2: Moderate harm	3 (12%)	60 (14%)
Level 3: Severe harm	0	7 (2%)
Level 4: Serious harm	0	1 (0.2%)

### Sensitivity analysis between prescribing models

The frequency of undocumented discrepancies associated with collaborative pharmacist prescribing was similar regardless of whether medications prescribed by a pharmacist required a medical countersignature. When a countersignature was not required, 921 medications were prescribed with 7 (0.8%) undocumented discrepancies, compared to 979 medications prescribed and 18 (2%) undocumented discrepancies when countersignature was required (RR 2.42, 95% CI 1.02–5.76). Discrepancies occurred in 7 versus 13 patients in the periods with and without countersignature respectively (RR 1.85, 95% CI 0.80–4.33) (Table 5).

**Table 5:** Comparison of outcomes: collaborative pharmacist prescribing model with medical officer countersignature required vs collaborative pharmacist prescribing model with no medical officer countersignature required

	Collaborative prescribing (medical countersignature)	Collaborative prescribing (no medical countersignature)	Relative risk (95% CI)
Total medications charted	979	921	2.42 (1.02–5.76)
Undocumented discrepancies (% of medications prescribed)	18 (2%)	7 (0.8%)	
Total patients	60	60	1.85 (0.80–4.33)
Patients with one or more undocumented discrepancy (% of patients)	13 (22%)	7 (12%)	

## Discussion

This study demonstrates that there were significantly fewer undocumented medication discrepancies in patients who received collaborative pharmacist prescribing compared to usual care within hospitals utilising EPS. The expert panel determined that undocumented discrepancies rarely had the potential to cause serious or severe patient harm in either group. These findings support the adoption of collaborative pharmacist prescribing models in hospitals utilising EPS, to optimise medication management, enhance patient safety and strengthen interprofessional collaboration.

The majority of published literature on collaborative prescribing in the hospital setting originates from Australia. In US hospitals, dependent prescribing models are more prevalent, while in the UK independent models are more commonly implemented. A systematic review identified 15 controlled studies related to collaborative and dependent prescribing models [1]. The review found that regardless of the pharmacist prescribing model used, compared to usual care, pharmacists prescribing was associated with reduced prescribing errors and medication omissions, pharmacists adherence to prescribing protocols and improved lipid and glucose control [1]. Similar to our study, international research on independent prescribing in the hospital setting has shown that the prescribing error rates for pharmacist prescribers are exceptionally low, ranging from 0.18 to 0.7%[2, 19, 34].

Our study addresses a gap in the literature regarding the impact of EPS on medication discrepancies in the collaborative pharmacist prescribing setting. At admission, 1% of medications prescribed by pharmacists had an undocumented discrepancy in our study, compared to rates of between 0.3% and 23% in paper-based settings [2, 9, 10]. Twenty one percent of patients who received collaborative pharmacist prescribing in our study had an undocumented discrepancy on admission, compared to 1.4%–9.5% reported in earlier studies in paper-based prescribing settings [7, 9, 15, 33]. This difference might reflect increased scope of pharmacist prescribing in our study, which included prescribing new medications, unlike some earlier studies which limited prescribing to pre-admission medications and venous thromboembolism prophylaxis.

A key strength of our study is the multi-site design across three tertiary hospitals within a statewide public healthcare system using the same EPS. Matching patients in both groups reduced the potential for unmeasured confounding. All undocumented discrepancies identified were independently reviewed for potential patient harm by the clinician panel, reducing potential bias, unlike some previous studies [9, 33, 35] which reviewed only a sample of discrepancies. Our panel included equal numbers of doctors and pharmacists, reducing risk of bias by a particular healthcare profession, overcoming limitations of previous studies [7, 15, 35]. Our study is also the first to assess potential harm from medication discrepancies with collaborative pharmacist prescribing using the validated HAMEC tool [31], which offers a standardised, reliable classification of medication-related harm. Our study is also the first of its kind to specifically assess how increased pharmacist autonomy, through the removal of a medical countersignature requirement, impacts the accuracy of pharmacist prescribing in the hospital setting.

Our findings align with existing research showing reductions in medication discrepancies or errors with collaborative pharmacist prescribing in hospitals using EPS [12, 14, 34]. Similar to our study, a pre-post study also found medication omissions were the most common error identified in both groups [14]. In our study, although both groups were matched on the number of pre-admission medications, collaborative pharmacist prescribing patients had more medications prescribed on admission. This may be because there were fewer medication omissions in the collaborative pharmacist prescribing group, and as a result, more medications were prescribed on admission.

Collaborative prescribing models have benefits beyond error reductions. In our study, all patients in the collaborative pharmacist prescribing group had a pharmacist-led medication history prior to prescribing, as required by the model, compared to only 12% in usual care. Having a pharmacist-led medication history available before prescribing has been shown to reduce prescribing discrepancies [27, 28, 36] consistent with the lower rate observed in the collaborative pharmacist prescribing group in our study. The impact of an early pharmacist-led medication history has also been compared to collaborative pharmacist prescribing in a paper-based setting. An Australian study found fewer prescribing errors occurred when doctors had access to a pharmacist-led medication history before prescribing (49.4%; 95% CI 42.5%-56.3%) compared to when they didn’t (61.4%; 95% CI 56.3%–66.7%) [10]. The lowest error rates in this study occurred when pharmacists collaboratively prescribed medications and had access to a pharmacist-led history prior to prescribing (3.5%; 95% CI 1.1%-5.8%) [10]. Our study adds to this existing evidence by showing the impact of having a pharmacist-led medication history prior to electronic prescribing on discrepancy rates. The collaborative clinical discussion between pharmacists and doctors, and the development of a shared medication plan, are central to this process [10]. Both the early pharmacist-led history, and inter-professional collaboration, likely contributed to the reduced discrepancies and potential harm observed in our study.

Our sensitivity analysis found that removing the medical countersignature requirement did not significantly impact prescribing discrepancies, with a non-significant trend towards fewer discrepancies. This may reflect increased prescriber experience and more cautious prescribing with the additional responsibility. Further research is warranted to confirm these hypotheses. Regardless, our findings indicate that pharmacists prescribe accurately and safely in collaborative models, and that removing the countersignature is not associated with increased prescribing discrepancies. This study provides evidence to support the adoption of collaborative pharmacist prescribing models in hospitals utilising electronic prescribing, demonstrating clear benefits in improving medication safety by reducing medication discrepancies and reductions in the potential for patient harm. Healthcare systems internationally may consider seeking legislative reform to expand pharmacists’ prescribing authority, alongside investment in electronic prescribing infrastructure, the development of competency frameworks, and targeted interprofessional training to foster collaborative decision making and ensure safe prescribing practices.

A limitation of our study is that medication discrepancies were identified retrospectively, and some may have been intentional changes that were not documented in the EMR. This highlights the importance for clear documentation of medication changes throughout the admission. Actual patient harm could not be determined in our study given inconsistent reporting of adverse drug events. Data collection was unblinded, as it was impossible to remove prescriber details from medication orders. However, the two independent pharmacist researchers who reviewed the discrepancies for validity, and the clinician panel were blinded during harm assessments to reduce bias. Although patients in both groups were successfully matched for indicators of medication-related harm, there may still have been unmeasured confounders that influenced our results. All patients in our study had a pharmacist-led medication history on admission to ensure consistent and accurate identification of discrepancies, aligning with the model’s requirements. While this approach enhances internal validity, it may limit generalisability of the results. The generalisability may also be constrained by the clinical setting (acute/general medical units), setting specific workflows or EPS functionality.

## Conclusion

Pharmacists working with doctors and patients to prescribe medications in hospitals utilising electronic prescribing systems is associated with significantly fewer medication discrepancies compared to usual care. The potential patient harm associated was also lower, although overall potential harm was low in both groups. Pharmacists prescribed just as accurately when the medical countersignature requirements were removed. Improvements in medication safety are likely attributed to pharmacists’ expertise, and the collaborative nature of the model and shared decision-making between the pharmacy and medical professions. These findings are timely and relevant as healthcare systems globally explore the expansion of pharmacists’ scope to improve access to care and support multidisciplinary models. With the global shift towards electronic prescribing systems, this study provides evidence to support the adoption of collaborative pharmacist prescribing models in hospitals with electronic prescribing systems to improve medication safety and reduce patient harm.

## Supplementary Information

Below is the link to the electronic supplementary material.

Supplementary file1 (DOCX 57 KB)

## Data Availability

De-identified participant data are available from the data custodian, SA Health, upon reasonable request. Access to the data is subject to relevant ethics and governance approvals and can only be granted upon request and approval by the data custodian, SA Health.
