# Supplementary material for: Evaluating medication discrepancies and harm: a matched cohort study of collaborative pharmacist prescribing in a statewide healthcare system using electronic prescribing
Source: Int J Clin Pharm. 2025 Aug 28;48(1):182–91. doi: 10.1007/s11096-025-01996-y (PMC12823733; doi:10.1007/s11096-025-01996-y)
Supplement: Supplementary file 1 — Supplementary file1 (DOCX 57 KB) [file 11096_2025_1996_MOESM1_ESM.docx]

**Supplementary Information**

**Article:** Evaluating medication discrepancies and harm: a matched cohort study of collaborative pharmacist prescribing in a statewide healthcare system using electronic prescribing

**Journal:** International Journal of Clinical Pharmacy

**Authors:** Hana Amer^1,2^ ORCiD: [0000-0002-0248-7241](https://orcid.org/0000-0002-0248-7241), Sally Marotti ^1,2,4^ ORCiD:[0000-0003-3098-6096](https://orcid.org/0000-0003-3098-6096), Joshua M Inglis^3,4^ ORCiD:[0000-0003-0486-9013](https://orcid.org/0000-0003-0486-9013), Imaina Widagdo^1^ ORCiD:[0000-0002-0669-0183](https://orcid.org/0000-0002-0669-0183), Sharon Goldsworthy^2^, Jacinta Johnson ^1,2^ ORCiD:[0000-0003-4786-022X](https://orcid.org/0000-0003-4786-022X), Lisa Kalisch Ellett ^1^ ORCiD:[0000-0001-5063-6128](https://orcid.org/0000-0001-5063-6128).

**Affiliations:**

^1^Quality Use of Medicines and Pharmacy Research Centre, UniSA Clinical and Health Sciences, University of South Australia, Adelaide, South Australia, Australia.

^2^SA Pharmacy, Adelaide, South Australia, Australia.

^3^ Department of Clinical Pharmacology, Flinders Medical Centre and Flinders University, Bedford Park, South Australia, Australia.

^4^School of Biomedicine, Faculty of Health Sciences, University of Adelaide, Adelaide, South Australia, Australia.

**Corresponding Author:**

**Hana Amer**

[hana.amer@mymail.unisa.edu.au](mailto:hana.amer@mymail.unisa.edu.au)

[hana.amer@sa.gov.au](mailto:hana.amer@sa.gov.au)

ORCiD: <https://orcid.org/0000-0002-0248-7241>

Quality Use of Medicines and Pharmacy Research Centre, UniSA Clinical and Health Sciences, University of South Australia, GPO Box 2471, Adelaide, SA, 5001, Australia

**Supplementary Figure 1.** Timeline of the commencement of collaborative pharmacist prescribing at each hospital site


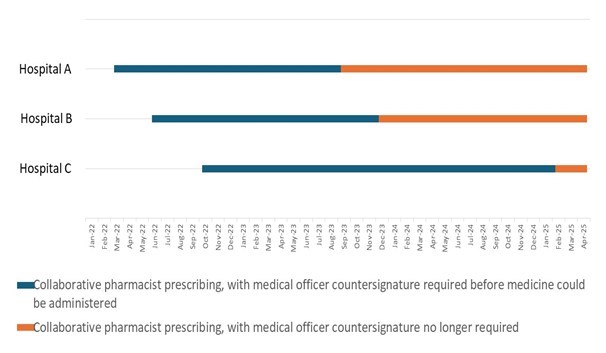


***Definition:*** *A Gantt chart illustrating the implementation timeline of collaborative pharmacist prescribing at each hospital site. Dark blue segments represent the period during which pharmacist prescribing required a medical countersignature before medication administration. Orange segments indicate the period during which a medical countersignature was no longer required. Figure created using Microsoft Office.*

**Supplementary Table 1.** Matching variables

|  | **Collaborative Prescribing Group**  N=120 | **Usual Care group**  N=120 | **p-value** |
| --- | --- | --- | --- |
| **Female sex, n (%)** | 67 (56%) | 66 (55%) | 0.897 |
| **Age at admission (median, IQR)** | 75 (65-85) | 78 (64-85) | 0.634 |
| **Triage category**^a^ | | | |
| **1 (Immediate)** | 7 (6%) | 9 (7.5%) | 0.636 |
| **2 (10 minutes)** | 46 (38%) | 41 (34%) |  |
| **3 (30 minutes)** | 59 (49%) | 60 (50%) |  |
| **4 (60 minutes)** | 8 (7%) | 8 (7%) |  |
| **5 (90 minutes)** | 0 | 2 (2%) |  |
| **Number of pre-admission medications, median (IQR)** | 11.5 (8-17) | 11 (7-15) | 0.69 |

^a^Australasian Triage Scale.

**Supplementary Table 2.** The Harm Associated with Medication Errors Classification (HAMEC): potential harm definitions

**Reproduced from CC BY-NC (Creative Commons Attribution-Noncommercial) licensed article:** Gates PJ, Baysari MT, Mumford V, Raban MZ, Westbrook JI. Standardising the Classification of Harm Associated with Medication Errors: The Harm Associated with Medication Error Classification (HAMEC). Drug Saf. 2019;42(8):931-9.<https://doi.org/10.1007/s40264-019-00823-4>.

| **Level** | **Reference** | **Description** |
| --- | --- | --- |
| **0** | No harm | No potential for patient harm, nor any change in patient monitoring, level or length of care required |
| **1** | Minor | There was potential for minor, non-life threatening, temporary harm that may or may not require efforts to assess for a change in a patient’s condition such as monitoring^a^. These efforts may or may not have potentially caused minimal increase in length of care (> 1 day) |
| **2** | Moderate | There was potential for minor, non-life threatening, temporary harm that would require efforts to assess for a change in a patient’s condition such as monitoring^a^, and additional low level change in a patient’s level of care^b^ such as a blood test. Any potential increase in length of care is likely to be minimal (< 1 day) |
| **3** | Serious | There was potential for major, non-life threatening, temporary harm, or minor permanent harm^c^ that would require a high level of care^b^ such as the administration of an antidote. An increase in the length of care of ≥ 1 day is expected |
| **4** | Severe | There was potential for life-threatening or mortal harm, or major permanent harm^c^ that would require a high level of care^b^ such as the administration of an antidote or transfer to intensive care. A substantial increase in the length of care of > 1 day is expected |

^a^Monitoring refers to the minimally intrusive observation of the patient’s condition over time. Observations are typically made for urine output, general level of consciousness, or vital signs including heart or breathing rate.

^b^Level of care refers to the degree of active treatments that are initiated in response to actual or potential change in the patient’s condition

^c^Permanent harm is such that, as a consequence of the drug event, the patient would require ongoing care, or experience an ongoing disability, beyond the index admission

**Supplementary Table 3.** Examples of undocumented discrepancies and their potential for patient harm as assessed by the expert panel

| **Level** | **Reference** | **Type of discrepancy** | **Order charted** | **Correct order** |
| --- | --- | --- | --- | --- |
| **0** | No harm | Omission | Omission | Multivitamin and minerals 1 tablet, orally, every morning |
| **1** | Minor | Omission | Omission | Fluticasone furoate - umeclidinium - vilanterol 100microgram-625microgram-25microgram/dose (TRELEGY ELLIPTA), 1 inhalation every morning |
| **2** | Moderate | Unnecessary medication | Ivabradine tablet, orally, 7.5mg, every night | Not taking ivabradine at home |
| **3** | Serious | Incorrect dose | valproate sodium enteric coated tablet, orally, 500mg every night | Valproate sodium enteric coated tablet, orally, 700mg every night |
| **4** | Severe | Omission | Omission | Warfarin (MAREVAN) tablet, orally, 8mg every night according to INR |
